# Female Pelvic Floor Dysfunction Continues to Negatively Impact Quality-of-Life during the COVID-19 Lockdown

**DOI:** 10.3390/jcm10051075

**Published:** 2021-03-05

**Authors:** Greta Lisa Carlin, Oliver Kimberger, Raffaela Morgenbesser, Wolfgang Umek, Heinz Kölbl, Klaus Bodner, Barbara Bodner-Adler

**Affiliations:** 1 Department of General Gynaecology and Gynaecologic Oncology, Medical University of Vienna, 1090 Vienna, Austria; greta.carlin@meduniwien.ac.at (G.L.C.); raffaela.morgenbesser@meduniwien.ac.at (R.M.); wolfgang.umek@meduniwien.ac.at (W.U.); heinz.koelbl@meduniwien.ac.at (H.K.); klaus.bodner@meduniwien.ac.at (K.B.); 2 Department of Anaesthesiology, Medical University of Vienna, 1090 Vienna, Austria; oliver.kimberger@meduniwien.ac.at; 3 Outcomes Research Consortium, Cleveland, OH 44195, USA; 4 Karl Landsteiner Institute of Special Gynaecology and Obstetrics, 3100 St. Pölten, Austria

**Keywords:** COVID-19 pandemic, lockdown, female pelvic floor dysfunction, pelvic floor related quality of life, pelvic organ prolapse, urinary incontinence

## Abstract

The COVID-19 pandemic led to dramatical changes in elective medical care. We analysed its impact on patients with female pelvic floor dysfunction during the 6 weeks of lockdown in Austria. A cross-sectional study was conducted: All 99 women who presented at the urogynaecologic outpatient clinic of the Medical University of Vienna with pelvic organ prolapse (POP) or urinary incontinence (UI) from December 2019 up to the lockdown in March 2020 were included and contacted. 97% of these women (96 participants) agreed to participate in the survey conducted to asses pelvic floor related quality of life (QoL) through telephone- interrogation. The mean age was 59 ± 14.8 years, the POP group consisted of 42 women while the UI group included 54 women. Most participants (83% of POP and 81% of UI cases) stated that their female pelvic floor dysfunction had remained equally relevant or had become even more significant during the lockdown. Associated symptoms and psychological strain also maintained their relevance during the lockdown (UI: *p* = 0.229; POP: *p* = 0.234). Furthermore, 97% of all interviewed women indicated to be strongly willing to continue their treatment. A generalised linear model regression revealed no clinical or demographic risk factors for psychological strain during the lockdown (*p* > 0.05). Our results demonstrate that women’s QoL remains significantly impaired by their pelvic-floor disorders even during a worldwide crisis such as COVID-19. Therefore, elective disciplines such as urogynaecology urgently require novel and innovative strategies for continued patient care even in times of a lockdown.

## 1. Introduction

As we write these lines, there is hope that the COVID-19-pandemic might be over by the end of 2021. The outbreak of COVID-19, caused by the new Severe Acute Respiratory Syndrome Coronavirus 2 (SARS-CoV-2) was located for the first time in Wuhan, China in December 2019 [1]. The COVID-19-pandemic will remain relevant in many respects, one of them being that it acted as a hard “stress-test” for the world’s health care systems. Resources had to be concentrated, patients’ paths within the health care systems were altered and, in many places, access to non-vital medical services was restricted altogether.

In Austria, the first patient with COVID-19 was diagnosed on 25 February 2020. By 15 March the number of COVID-19 patients had risen to 860 with exponentially increasing infections rates [2,3,4]. At the same time, neighbouring Italy experienced an overburden of the healthcare system with dramatic consequences. Hoping to prevent a similar course from occurring in Austria, the government imposed a strict lockdown beginning on 16 March 2020. All non-essential shops and business were closed as were all educational institutions [5]. Hospitals suspended outpatient clinics for non-emergency patients as well as cancelled elective surgeries on short notice. Neither medical staff nor patients knew when appointments and surgeries would be rescheduled, because it was not clear how long the lock-down would last. Due to the social restrictions aiming to flatten the infection-curve many patients were left without care. Previous studies have demonstrated how patients’ quality of life has suffered significantly across many medical fields, such as surgery and urology [6,7,8,9,10].

It is well known that pelvic floor dysfunctions adversely affect the quality of life including the social, emotional, and sexual well-being [11]. Patients affected by pelvic floor dysfunction, like urinary incontinence or pelvic organ prolapse, usually attend specific urogynaecologic facilities frequently, because these conditions—even though not life-threatening—cause great discomfort and restrictions in daily life and thus require regular follow-up care [12]. Thus far, data about the effect of the COVID-19-lockdown and its consequences on pelvic floor dysfunction related quality of life in this group of patients have been very limited.

We set out to answer the question of how much and in which way, patients with female pelvic floor dysfunction were affected by the first lockdown.

The primary aim of this study was to determine female pelvic floor dysfunction related quality of life during the COVID-19 lockdown. The secondary aim of this study was to gain insights into the difference of patient’s symptoms and psychological strain before vs. during lockdown. We hypothesized that female pelvic floor dysfunction would further add to the negative impact on patients’ quality-of-life already caused by the COVID-19 lockdown and its socioeconomic consequences.

## 2. Materials and Methods

All 99 patients, who visited the urogynaecologic outpatient clinic of the Medical University of Vienna due to any type of urinary incontinence (UI) or pelvic organ prolapse (POP) in the time between December 2019 and March 2020 (until the lockdown: dated with 16.3.2020 in Austria), were eligible for this cross-sectional study. 96 of these 99 women (97%) accepted to be interviewed by telephone and were included in the study. The participants were classified into two groups: The first group consisted of patients with verified urinary incontinence (*n* = 54), which included patients with symptomatic stress UI (SUI), mixed UI and overactive bladder (OAB) into the study. OAB was defined as OAB wet (urge incontinence) or OAB dry (without incontinence). In patients with mixed UI the dominant complaint was chosen as study diagnosis. The second group consisted of patients with symptomatic pelvic organ prolapse (*n* = 42). In patients with a combination of symptomatic urinary incontinence and prolapse the dominant complain, i.e., the complaint the patients’ treatment plan focused on, was used as study diagnosis. Clinical information and confirmation of the female pelvic floor dysfunction diagnosis including a comprehensive physical examination, staging via Pelvic Organ Prolapse Quantification system (POP-Q system) and clinical stress-test with 300 mL of saline accordioning to the International Continence Society (ICS) [13,14] were obtained from the patients’ electronic hospital chart. All patient records were anonymized and de-identified prior to analysis. Only non-emergency patients and patients with planned elective reconstructive pelvic floor procedures who were able to understand German were included. Emergency cases and all non-urogynaecologic issues, as well as patients with language difficulties were excluded from this survey. The criteria for emergency cases used by our institution included: postoperative concerns or complications, new onset of genitourinary fistula, acute urinary retention, acute interstitial cystitis, pessary complications, mesh complication and acute urethral or vaginal mass (other than prolapse). All cases with these complaints or diagnosis were excluded from the study as they were handled as emergency cases with an emergency in door appointment at our clinic.

The study was approved by the ethics committee of Medical University Vienna (EK No 1527/2020).

96 women with known pelvic floor dysfunction (PFD) answered our survey by use of telephone-interrogations about the three domains: bladder, prolapse, and sexuality six weeks after the beginning of the lockdown in Austria. Two staff members of the urogynaecologic team (GLC and RM) conducted the interview during the first nation-wide lock-down between 21 and 24 of April 2020. The interviewers filled out the survey after complete response by the participants in an estimated mean time of 10 min. In all cases, ethical research standards were complied with by providing information on the project and requesting consent to participate.

At this point in time no validated questionnaires addressing pelvic floor related QoL and the Covid19 pandemic were available, therefore, we developed a novel questionnaire in German, based on the basic structure of the validated version of the German pelvic floor questionnaire by Baesslar et al. [15]. Our questionnaire consisted of 13 questions in four sections. The four sections of the questionnaire addressed general health, urinary incontinence, pelvic organ prolapse, and sexuality. Patients´ answers were scored from zero (=not at all) to two points (=a lot) and with additional subjective scores from 0 (=no relevance) to 10 (=highest relevance) for relevance of the symptom to the patient. A higher score reflected a greater subjective relevance of the respective female pelvic floor dysfunction. The main focus of questions was regarding if and how patients’ pelvic floor related QoL had changed during the lockdown. The complete questionnaire is presented as Supplementary Figure S1.

### Statistical Analysis

Chi-square was used for the comparison of categorical variables between the two groups (incontinence versus prolapse group) and Student’s *t*-test for continuous variables. A paired *t*-test was used for analysis of difference of variables before and after lockdown. The normality of data was visually assessed with QQ plots and Shapiro Wilk test. The average score of the three domains (incontinence, prolapse, sexual function) in the questionnaire was reported as mean and standard deviation (sd). For correlation analysis Spearman test was used with correlation coefficient. A generalised linear model was performed to identify independent parameters associated with quality of life related pelvic floor domains. A *p*-value < 0.05 was considered statistically significant. The SPSS system (IBM, Armonk, NY, USA, Version 23) was used for the calculations.

## 3. Results

In total, 99 patients with pelvic floor dysfunction were seen in our urogynaecology outpatient clinic between December 2019 and March 2020, we were able to contact 96 patients, who consented to answer the telephone-questionnaire (42 with symptomatic POP ≥ stage 2 and 54 with verified UI). The patients’ mean age was 59 (±14.8) years, ranging from 26 to 87 years. No statistically significant demographic differences could be observed between the two groups (*p* > 0.05). Patients’ characteristics for the whole study group are presented in Table 1.

65% of interviewed patients (63/96) stated that they experienced negative changes of their QoL during lockdown. 30 patients (31%) reported only some negative changes, while 33 patients (34%) experience a lot of negative changes (Table 2).

In the UI group 29 of 54 patients (54%) reported that their symptoms remained equally relevant during the lockdown, while 15 women (28%) reported that this specific problem gained even more relevance during the lockdown, as can be viewed in Table 3.

Similarly, results were obtained from the POP group: 24 of 42 patients (57%) with symptomatic POP reported that their prolapse issue was still equally relevant during the lockdown and for 11 women (26%) it had become even more relevant, as seen in Table 4.

Furthermore, the symptom burden the women suffered from also remained equal, as mean scores of relevance of the female pelvic floor dysfunction did not statistically significant differ between before compared to during lockdown (UI Cases: *p* = 0.229 and POP cases: *p* = 0.234) (Table 3 and Table 4).

Mean scores of relevance of sexuality remained similar before to during the lockdown (mean score for sexual function: 3.96 ± 2.380 versus 4.11 ± 2.383; *p* = 0.228) (Table 5).

Furthermore, 97% of all interviewed women indicated to be strongly willing to continue their treatment, notwithstanding the risk of possible exposure to COVID-19 when attending the urogynaecologic outpatient clinic.

In addition, a significant positive correlation could be observed between POP-Q stage and prolapse affliction during lockdown, signifying more symptom burden from prolapse in women with advanced prolapse stage (correlation coefficient = 0.0001).

When controlled for age, parity, menopausal status, BMI and smoking via generalised linear model, none of the above-mentioned parameters turned out to be an independent risk factor for psychological strain during the lock-down (*p* > 0.05).

## 4. Discussion

82% of patients with urinary incontinence and 83% of patients with pelvic organ prolapse stated that their pelvic floor dysfunction remained equally relevant or even gained significance during the lockdown. Female pelvic floor dysfunction symptoms as well as psychological strain before compared to during lockdown also remained unchanged, meaning that the burden on the patients remains equal even during these exceptional circumstances caused by COVID-19. Furthermore, nearly all women (97%) expressed a strong desire to continue their treatment, regardless of the fear or possibility of contagion when attending the hospital.

Symptoms-severity of patients with pelvic floor dysfunction remains relevant even in times of medical crisis like COVID-19 lockdown. Our impression was that the temporary closure of the urogynaecologic outpatient clinic as well as the cancellation of all elective pelvic floor surgeries left many patients without care, which likely caused the negative impact on their quality of life we demonstrated in this study. This is further confirmed by the fact that all patients, who were invited to attend our clinic at a later date, were willing to resume their treatment as soon as possible. Especially, patients with planned surgical appointments requested these to be undertaken as soon as possible.

Brusciano et al. reported similar findings, demonstrating that patients with faecal incontinence, pain and functional constipation, felt great motivation to undergo pelvic floor rehabilitation even in times of COVID-19 pandemic [6]. In line with our findings, the authors showed that their patients were concerned that discontinuation of treatment could affect their quality of life during the crisis.

The observation of our study as well as the results of Brusciano et al. confirm how pelvic-floor related quality of life is tightly connected to special treatment offers in this group of patients. New strategies to maintain continuity in patient care even under unexpected circumstances are therefore urgently required.

### 4.1. Limitations and Strengths of the Study

A strength of this study is the fact that it was conducted at the time of the first national COVID-19 lockdown and thus reflects a real-time experience of the participating patients.

We are aware of limitations of our study. As this was a cross-sectional study, we could not analyse patients’ attitude over a period of time. However, we tried to choose the most representative point in time: 6 weeks after the start of the first lockdown in Austria. We are aware that 6 weeks of lockdown could be a short time period for deterioration in perceived importance of symptoms and severity to occur, therefore, a follow-up of our study group is planned. Furthermore, we conducted a telephone-interview with the aim to reach as many representative cases with PFDs as possible, thus no randomization was performed, nor control group included. We had to develop our own questionnaire, because we were the first to investigate specific symptoms of pelvic floor dysfunction and their impact on QoL during the COVID-19 lockdown. This questionnaire was novel and as such not yet validated, posing a further limitation to this study. In addition, as the questionnaire compares patients’ current symptom severity perception to their earlier experience, recall bias cannot be excluded. Lastly, the statistical power of our study is also limited. Nevertheless, as the response rate to this study was 97%, we believe to be able to offer an informative view into patients’ pelvic-floor related QoL during the first lockdown in Austria.

### 4.2. Strategies for the Future

We have learned from our survey that pelvic-floor related quality of life remains impaired even in times of crisis. As individual well-being has great importance, we should not leave this patient group alone, least of all during such critical situations. We are convinced that the implementation of telemedicine, through telephone and video calls, during such a crisis is of outstanding significance for this group of patients. These platforms can be useful tools for the structured guidance of patients through their behavioural and lifestyle changes and can be used for patient education. The importance of this topic is also emphasized by Grimes et al. and Serna-Gallegos et al. who published how one can implement virtual patient care by use of telemedicine, trying to present strongly lacked guidance for urogynaecology patient management during pandemics as COVID-19 [7,16]. Moreover, Gray et al. pointed out how the resumption of urogynaecologic services could strain the system with increased demand and should therefore be well organised [17].

## 5. Conclusions

Subjective well-being is of great importance and the basic right of each individual. Our results clearly showed that symptoms-severity of patients with pelvic floor disorders remained relevant even in terms of crisis. Furthermore, pelvic-floor related quality of life is strongly connected to special treatment offers and we could also demonstrate the importance of allowing patients with female pelvic floor dysfunction to express themselves and their needs in times of quarantine and social distancing. Therefore, disciplines such as urogynaecology urgently require novel and innovative strategies for ambulatory and non-emergency patient care throughout times like the COVID-19 era.

## Figures and Tables

**Table 1:** Patients characteristics of interviewed women with pelvic floor dysfunctions.

	*n* = 96 (100%)
Parameter	*n* (%) or Mean (±sd ^1^)
Age (years)	58.9(±14.8)
Age at menopause (years)	53.3 (±2.9)
BMI ^2^ (kg/m^2^)	26.9 (±3.76)
Hypertension	57 (59%)
Smoking	17 (1%)
Parity	1.87 (±1.34)
PFD ^3^ treatment	
Conservative	44 (46%)
Planned pelvic floor surgery	52 (54%)

^1^ sd = standard deviation; ^2^ BMI = body mass index; ^3^ PFD = pelvic floor disorder.

**Table 2:** Negative changes of quality of live during COVID-19 lockdown.

	*n* = 96 (100%)
Negative Changes of QoL ^1^ during Lockdown	*n* (%)
Not at all	33 (34%)
Some	30 (31%)
A lot	33 (34%)

^1^ QoL = quality of life.

**Table 3:** Changes in relevance and affliction of urinary incontinence symptoms during COVID-19 lockdown.

Cases with UI ^1^ (*n* = 54)		
Parameter	*n* (%) or Mean (±sd ^2^)	*p*-Value ^3^
How relevant did the UI affliction feel during the COVID-19 lockdown?		
Less relevant	10 (19%)	
Equally relevant	29 (54%)	
More relevant	15 (28%)	
Scale of UI symptom affliction (0–10) before lockdown	5.74 (±1.91)	
Scale of UI symptom affliction (0–10) during lockdown	5.24 (±2.70)	0.229

^1^ UI = urinary incontinence; ^2^ sd = standard deviation; ^3^ *p*-value for paired *t*-test.

**Table 4:** Changes in relevance and affliction of pelvic organ prolapse symptoms during COVID-19 lockdown.

Cases with POP ^1^ (*n* = 42)		
Parameter	*n* (%) or Mean (±sd ^2^)	*p*-Value ^3^
How relevant did the POP affliction feel during the COVID-19 lockdown?		
Less relevant	7 (17%)	
Equally relevant	7 (57%)	
More relevant	11 (26%)	
Scale of POP symptom affliction (0–10) before lockdown	5.98 (±2.30)	
Scale of POP symptom affliction (0–10) during lockdown	5.67 (±2.12)	0.234

^1^ POP = pelvic organ prolapse; ^2^ sd = standard deviation; ^3^ *p*-value for paired *t*-test.

**Table 5:** Influence of Covid19 lockdown on sexual life.

	*n* = 96 (100%)	
Parameter	*n* (%) or Mean (±sd ^1^)	*p*-Value ^2^
Has the COVID-19 lockdown influenced your sexual life?		
Not at all	78 (81%)	
Some	14 (15%)	
A lot	4 (4%)	
Scale of relevance of sexual life (0–10) before lockdown	3.96 (±2.38)	
Scale of relevance of sexual life (0–10) during lockdown	4.11 (±2.38)	0.228

^1^ sd = standard deviation; ^2^ *p*-value for paired *t*-test.

## Supplementary Materials

The following are available online at https://www.mdpi.com/2077-0383/10/5/1075/s1, Figure S1: Questionnaire COVID-19 lockdown and pelvic floor issues.
