# Supplementary material for: Female Pelvic Floor Dysfunction Continues to Negatively Impact Quality-of-Life during the COVID-19 Lockdown
Source: J Clin Med. 2021 Mar 5;10(5):1075. doi: 10.3390/jcm10051075 (PMC7961535; doi:10.3390/jcm10051075)
Supplement: Supplementary file 1 [file jcm-10-01075-s001.pdf]

Figure S1: Questionnaire Covid-19 lockdown and pelvic floor issues

General:

1. How has the Covid-19 lockdown influenced your quality of life?
   - Not at all
   - Quite a bit
   - A lot
2. On a scale of 0-10 what relevance did you give your urogynecological issue BEFORE the Covid-19 lockdown? no relevance (0) - highest relevance (10)
3. On a scale of 0-10 what relevance did you give your urogynecological issue DURING the Covid-19 lockdown? no relevance (0) - highest relevance (10)
4. What urogynecological issue do you suffer from?
   - Urinary incontinence (continue with section "urinary incontinence")
   - Prolapse (continue with section "prolapse")

Urinary incontinence:

5. Did you suffer from urinary incontinence during the Covid-19 lockdown?
   - no
   - yes
6. How relevant did your urinary incontinence affliction feel during the Covid-19 lockdown?
   - less relevant during the Covid-19 lockdown than before
   - just as relevant during the Covid-19 lockdown as before
   - more relevant during the Covid-19 lockdown than before
7. How did your urinary incontinence afflict you during the Covid-19 lockdown?
   - Not at all
   - Quite a bit
   - A lot

Prolapse:

8. Did you suffer from prolapse-symptoms during the Covid-19 lockdown?
   - No
   - Yes
9. How relevant did your prolapse feel during the Covid-19 lockdown?
   - less relevant during the Covid-19 lockdown than before
   - just as relevant during the Covid-19 lockdown as before
   - more relevant during the Covid-19 lockdown than before
10. How did your prolapse afflict you during the Covid-19 lockdown?
    - Not at all
    - Quite a bit
    - A lot

Sexual function:

11. Has the Covid-19 lockdown influenced your sexual life?
    - Not at all
    - Some
    - A lot
12. On a scale of 0-10 what relevance did you give your sexual life BEFORE the Covid-19 lockdown?
    no relevance (0) - highest relevance (10)
13. On a scale of 0-10 what relevance did you give your sexual life DURING the Covid-19 lockdown?
    no relevance (0) - highest relevance (10)
